# Unravelling the genetic causes of mosaic islet morphology in congenital hyperinsulinism

**DOI:** 10.1002/cjp2.144

**Published:** 2019-10-29

**Authors:** Jayne AL Houghton, Indraneel Banerjee, Guftar Shaikh, Shamila Jabbar, Thomas W Laver, Edmund Cheesman, Amish Chinnoy, Daphne Yau, Maria Salomon‐Estebanez, Mark J Dunne, Sarah E Flanagan

**Affiliations:** ^1^ The Genomics Laboratory, Royal Devon and Exeter Foundation Hospital Exeter UK; ^2^ Molecular Genetics, Institute of Biomedical and Clinical Science University of Exeter Medical School Exeter UK; ^3^ Department of Paediatric Endocrinology Royal Manchester Children's Hospital Manchester UK; ^4^ Faculty of Biology, Medicine and Health The University of Manchester Manchester UK; ^5^ Department of Paediatric Endocrinology Royal Hospital for Children Glasgow UK; ^6^ Department of Paediatric Pathology Royal Manchester Children's Hospital Manchester UK

**Keywords:** mosaic disease, congenital hyperinsulinism, *ABCC8*, pancreas

## Abstract

Congenital hyperinsulinism (CHI) causes dysregulated insulin secretion which can lead to life‐threatening hypoglycaemia if not effectively managed. CHI can be sub‐classified into three distinct groups: diffuse, focal and mosaic pancreatic disease. Whilst the underlying causes of diffuse and focal disease have been widely characterised, the genetic basis of mosaic pancreatic disease is not known. To gain new insights into the underlying disease processes of mosaic‐CHI we studied the islet tissue histopathology derived from limited surgical resection from the tail of the pancreas in a patient with CHI. The underlying genetic aetiology was investigated using a combination of high depth next‐generation sequencing, microsatellite analysis and p57kip2 immunostaining. Histopathology of the pancreatic tissue confirmed the presence of a defined area associated with marked islet hypertrophy and a cytoarchitecture distinct from focal CHI but compatible with mosaic CHI localised to a discrete region within the pancreas. Analysis of DNA extracted from the lesion identified a *de novo* mosaic *ABCC8* mutation and mosaic paternal uniparental disomy which were not present in leukocyte DNA or the surrounding unaffected pancreatic tissue. This study provides the first description of two independent disease‐causing somatic genetic events occurring within the pancreas of an individual with localised mosaic CHI. Our findings increase knowledge of the genetic causes of islet disease and provide further insights into the underlying developmental changes associated with β‐cell expansion in CHI.

## Introduction

Congenital hyperinsulinism (CHI) is characterised by dysregulated insulin secretion which can lead to life‐threatening hypoglycaemia. Patients who do not respond to medical therapy may require a pancreatectomy with the extent of the resection determined by the genetic aetiology 1.

Three sub‐classes of CHI exist, which are associated with distinct histological descriptions (see below) and include diverse islet endocrine cell types 1, 2, 3. CHI is distinctly different from pancreatic tumours such as insulinomas, which are derived from expansion of insulin producing cells 4. The pathology of diffuse CHI (CHI‐D) encompasses the entire endocrine pancreas and can result from dominant or bi‐allelic recessive mutations in one of the known disease‐causing genes. Islets in CHI‐D appear within the context of intact lobular architecture and are associated with nucleomegaly in some cells 2.

In contrast, focal CHI (CHI‐F) occurs as a localised region of adenomatous hyperplasia of abnormal β‐cells with limited clonal expansion but without neoplastic transformation. In CHI‐F unmasking of a paternally inherited recessively acting *ABCC8/KCNJ11* mutation and the concurrent loss of an imprinted region by paternal uniparental disomy (UPD) upstream of the genes confers a growth advantage to the cell leading to the development of a focal lesion which over‐secretes insulin 5, 6. Unlike pancreatic tumours such as insulinomas, focal CHI lesions also contain glucagon‐, somatostatin‐, and pancreatic polypeptide‐secreting cells 3, 4.

More rarely, mosaic histology is described where there are hyperplastic islets, often in a discrete lobe of the pancreas with shrunken/condensed islets existing outside the affected lobular region 3. Mosaic CHI differs from that of the focal disease because of the absence of adenomatous hyperplasia. The presence of Localised Islet Nuclear Enlargement (LINE) within the affected lobe of the pancreas can also signify mosaicism 3, 7. The underlying genetic cause(s) of mosaic‐CHI are unknown.

We describe the genetic and histopathological findings of a case with mosaic‐CHI, providing new insights into the disease mechanisms of this disorder.

## Materials and methods

The female proband was born at 39 weeks gestation with a birth weight of 4.01 kg to unaffected, non‐consanguineous parents. She developed hypoglycaemic seizures within 24 h of birth requiring 15% dextrose and glucagon infusion. CHI was confirmed (plasma glucose 1.1 mmol/l [19.8 mg/dl], insulin 224 pmol/l) and glycaemic control was achieved with partial response to diazoxide (15 mg/kg/day), followed by second line treatment with Octreotide (30 mcg/kg/day). Syndromic causes of hyperinsulinism were excluded by clinical genetic review. As the frequency of hypoglycaemia increased, at 2.7 years the patient underwent 18^F^DOPA PET‐CT scanning which identified a focus of increased isotope activity. As the patient was only partially responsive to treatment with diazoxide and required high dose Octreotide by subcutaneous injections with significant risk of side effects, partial pancreatic surgery was considered as the therapy of choice. The lesion within a pancreatic lobule was removed by laparoscopic surgery which effected cure. The patient underwent a fast following pancreatectomy with adequate ketogenesis and absence of hypoglycaemia. Pre‐feed plasma glucose was tested by home glucose monitoring for 6 months after surgery; no episodes of hypoglycaemia were recorded. There have been no concerns regarding clinical suspicion of hypoglycaemia 1 year after surgery.

The study was conducted in accordance with the Declaration of Helsinki with informed consent obtained from the parents (REC 07/H1010/88).

### Histopathology

H&E staining was used to exclude insulinoma as a cause for hyperinsulinism. Immunohistochemistry for insulin, glucagon, somatostatin and p57kip2 expression was performed on formalin‐fixed paraffin‐embedded (FFPE) pancreatic tissue as described previously 8. Comparative quantification of islets was performed using tissues from CHI‐F (*n* = 12), CHI‐D (*n* = 6) and healthy controls (*n* = 4) 8.

### Genetics

Targeted next generation sequencing of 14 genes, (*ABCC8*, *KCNJ11*, *GLUD1*, *HADH*, *GCK*, *HNF4A*, *HNF1A*, *SLC16A1*, *CACNA1D*, *KDM6A*, *KMT2D*, *PMM2*, *INSR1*, *TRMT10A*) known to cause isolated or syndromic CHI was undertaken using DNA extracted from the patient's leukocytes and FFPE pancreatic tissue excised from the lesion and surrounding normal tissue 9. Coding mutations in the *MEN1* gene were excluded by Sanger sequencing of leukocyte DNA.

Following the identification of a mutation, Sanger sequencing of *ABCC8* exon 37 (NM_001287174.1) was performed on leukocyte and buccal cell DNA from the patient and leukocyte DNA from the parents 10.

Loss‐of‐heterozygosity was investigated by analysis of 12 microsatellites spanning Chr11p15 using DNA extracted from the pancreatic lesion, surrounding normal tissue and leukocytes from the patient and parents as previously described 10.

## Results

### Mosaic‐CHI islet profile and LINE

**Figure 1:**
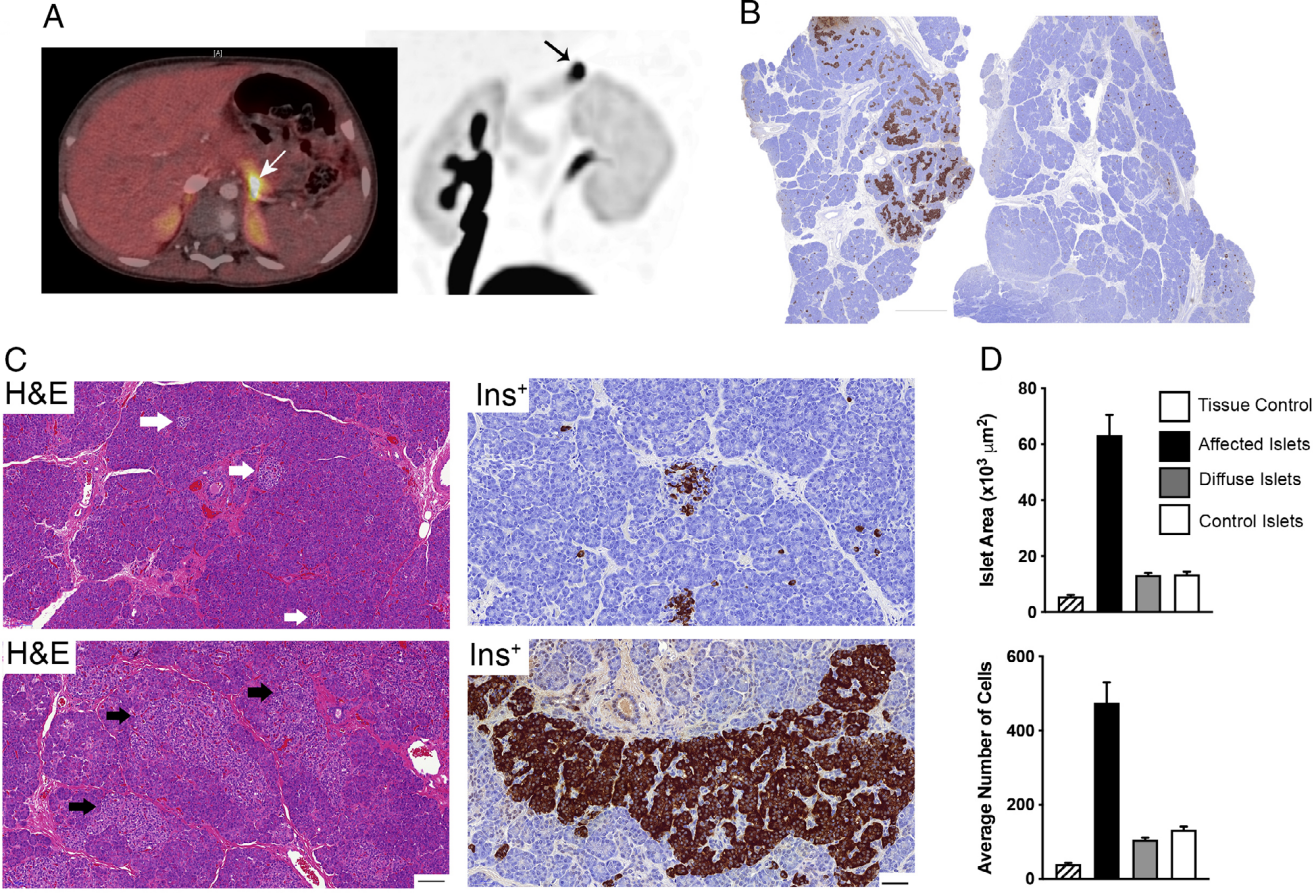
Lobular organisation of mosaic CHI. (A) ^18^F‐DOPA PET‐CT image scans reveal a discrete area with high uptake of tracer in the tail of the pancreas, indicated by the arrows in both superimposed PET/CT (left) and maximum intensity projection images (right). (B) Different parts of the resected pancreas stained for immunoreactivity to insulin. Note the abnormally high level of insulin‐producing cells localised only to a particular region of the pancreas which is not observed elsewhere. Scale bar, 2 mm. (C) H&E and insulin (Ins^+^) immunohistochemistry to compared islets from the healthy part of the pancreas (upper images, white arrows in the H&E figure) with those from the unaffected regions (lower images, black arrows in the H&E figure). The most noticeable pathological feature of the islets from within the affected region is hyperplasia leading to a marked increase in size and structure. Scale bars; H&E 100 μm: insulin 50 μm. (D) Quantitative analysis of abnormal islets compared to tissue controls, diffuse CHI islets (*n* = 6 cases) and control human islets (*n* = 12 cases). Note how abnormal islets have significantly increased surface areas and islet cell numbers, *p* < 0.0001.

^18^F‐DOPA PET‐CT scanning revealed the presence of a single 22.5 mm^2^ area of hyperactive tissue within the pancreas (Figure 1A). Histological analysis demonstrated abnormal tissue architecture confined to a lobule (Figure 1B). Unlike most CHI‐F tissues, islet cells were not enriched and localised to a discrete anatomical region or focus circumscribed by a well‐defined capsule, but instead were interspersed with exocrine components (Figure 1C). The distribution of exocrine and endocrine tissues was suggestive of mosaic CHI.

**Figure 2:**
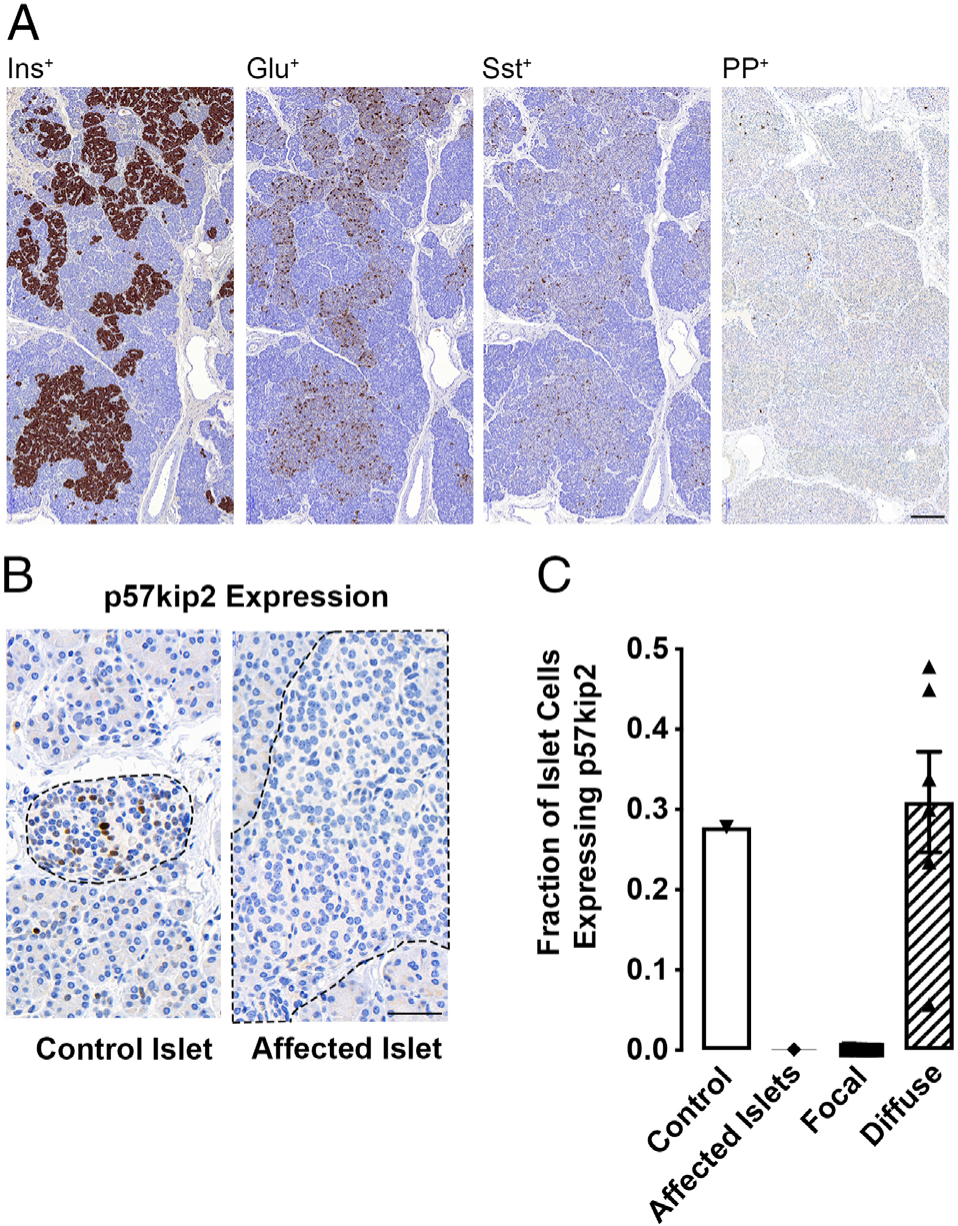
Profile of islet hormones and p57kip2 expression in CHI tissues. (A) The expression of insulin (Ins^+^), glucagon (Glu^+^), somatostatin (Sst^+^) and pancreatic polypeptide (PP^+^) in islet cells from the affected region of the pancreas, scale bar 200 μm. (B) Representative images of islet (indicated by the dotted regions) p57kip2 expression in the mosaic tissue from both the control and affected regions (scale bar, 50 μm). (C) Quantitative expression of p57kip2 in islet cells from the mosaic tissue (affected and control regions) compared to focal CHI lesions (*n* = 12 cases) and diffuse islets (*n* = 6 cases).

The islets expressed insulin, glucagon, somatostatin and pancreatic polypeptide, indicating the diversity of different endocrine cells without preferential insulin enrichment in contrast to CHI‐F tissue (Figure 2A). The presence of other islet endocrine cells also excluded insulinoma as a possible cause of disease 4. Islet surface area was 4.8‐fold larger than age‐matched control islets and islets from CHI‐D tissue, *p* < 0.0001 (Figure 1D), indicating a clear distinction from CHI‐D. The histology was typical of mosaic CHI with affected islets being irregular in shape, hyperplastic and containing more cells than controls or CHI‐D islets (*p* < 0.0001) (Figure 1D). In contrast, islets outside the lobule were shrunken compared to controls (*p* < 0.0001) and contained fewer cells (*p* < 0.0001) (Figure 1D). Mosaic islets contained numerous cells with enlarged nuclei (average nuclear surface area 71.4 ± 4 μm^2^ [*n* = 201 cells], *p* < 0.001) which were significantly larger than control islet cells (34 ± 4 μm^2^ [*n* = 195]) but smaller than islet cells from CHI‐D with nucleomegaly (115 ± 5.3 μm^2^ [*n* = 103]). These criteria, and the appearance of shrunken cells with dense nuclear crowding in islet cells from outside the affected region (Figure 1C), are consistent with previous descriptions of LINE and mosaic CHI disease 3.

### Loss of p57Kip2 expression

p57kip2 staining demonstrated clear nuclear reactivity in 28–31% of surface islet cells from control or CHI‐D tissue, but not in islet cells examined within the impaired lobular domain (Figure 2B,C). This suggests that, in mosaic‐CHI, as with CHI‐F, impaired expression of the cell cycle repressor p57kip2 correlates with abnormal islet cell development leading to inappropriate β‐cell expansion.

### Two post‐zygotic genetic events within the lesion

Microsatellite analysis showed mosaic maternal loss‐of‐heterozygosity (with an average maternal peak height reduction of 27%) (see supplementary material, Figure S1) confined to the affected pancreatic tissue which spanned a minimal region of 3.1 Mb (Chr11:1,445,456‐17,567,129), extending across the differentially methylated region and *ABCC8*. Dosage analysis was consistent with loss‐of‐heterozygosity resulting from UPD rather than a deletion.

Targeted next‐generation sequencing, followed by confirmatory Sanger sequencing, identified a mosaic p.(Glu1507Lys); c.4519G>A, *ABCC8* variant (119/419 reads [28%]) in the affected tissue (see supplementary material, Figure S1). This variant was not called in the normal pancreatic tissue, buccal cell DNA nor the peripheral leukocytes of the patient and parents. It was not possible to deduce whether the p.(Glu1507Lys) variant had arisen on the maternal or paternal chromosome as no informative variants were detected on sequencing which would have allowed for the mutation to be phased.

## Discussion

Early identification of the underlying pathobiology of CHI is crucial for guiding treatment at an early stage. Although there are key diagnostic criteria for CHI‐D and CHI‐F, up to 10% of cases have atypical histology which have been variably described as mosaic CHI or LINE. An understanding of the pathology in such cases is limited by the variability in phenotype and infrequent access to pancreatic tissue. Here, we have identified a novel genetic cause of CHI in a patient who has the typical histological features of mosaic CHI 3.

The islet hypertrophy and cytoarchitecture observed in mosaic CHI is distinct from that of CHI‐F where loss of p57kip2 leads to β‐cell hyperplasia and the formation of a discrete mass of islet cells, often contained within a capsule demarcating the lesion 11. In mosaic CHI, hyperplastic islets are constrained to a lobe of the pancreas where they appear enlarged in size when compared to islets outside the affected lobule(s) 3. These criteria were observed within the affected pancreatic lobule in our patient, alongside the presence of shrunken islets with nuclear crowding outside the lesion, typifying the diagnosis of mosaic CHI. This histopathological description is also compatible with LINE 3, 7, where nuclear enlargement is considerably smaller than irregular nucleomegaly observed in patients with diffuse CHI 8.

Whilst the organisation of islets in mosaic CHI patients is distinct from CHI‐D and CHI‐F, the underlying genetic cause(s) of disease have remained unknown. Here, we report how islet mosaicism resulted from a mosaic *ABCC8* mutation, p.(Glu1507Lys), which is reported as dominantly acting 12, together with mosaic paternal UPD of chromosome 11p15 within the pancreatic lesion.

Whilst the underlying genetic aetiology in our patient is similar to that described in CHI‐F, whereby paternal UPD unmasks a recessively acting germline or somatic mutation 13, to our knowledge two independent somatic events isolated to the pancreatic tissue have not been reported in patients with CHI to date. Furthermore, a difference between the genetics in our case and CHI‐F is the presence of a dominant, rather than recessively acting, K‐ATP channel mutation. As heterozygosity for the p.(Glu1507Lys) mutation is sufficient to cause over‐secretion of insulin from the β‐cell, and given that the parent of origin for the chromosome harbouring the mutation was not determined, it has not been possible to deduce the temporal sequence of genetic events and how this has contributed to abnormal tissue expansion. If the mutation had arisen on the paternal chromosome it is possible that it was present in cells outside the lesion but at a level below our limit of detection (i.e. <2% using a mosaic variant caller for targeted next generation sequencing of DNA from leukocytes and unaffected pancreas and <10% for Sanger sequencing of buccal DNA) which only became detectable following a UPD event within the lesion.

Whilst we report a novel aetiology for mosaic histological disease, given the heterogeneous nature of this condition it is possible that other genetic mechanisms for mosaic CHI exist. Based on our findings we recommend in‐depth molecular genetic testing of DNA extracted from affected pancreatic tissue as a way of identifying novel disease‐causing mechanisms of CHI.

In conclusion, we describe two disease‐causing somatic events within the pancreas of an individual with CHI and illustrate how studying pancreatic DNA is crucial for improving knowledge on the underlying mechanisms of mosaic‐CHI.

## Author contributions statement

JALH, IB, MJD and SEF conceived the study. JALH, IB, GS, SJ, TWL, EC, AC, DY, MSE, MD and SEF carried out experiments and analysed the data. IB, MJD and SEF drafted the manuscript which was reviewed and approved by all authors. JALH and IB, and MJD and SEF, contributed equally to this study.

## Supporting information


**Figure S1.** Results of microsatellite and sequence analysis
