# Supplementary material for: Unravelling the genetic causes of mosaic islet morphology in congenital hyperinsulinism
Source: J Pathol Clin Res. 2019 Oct 29;6(1):12–6. doi: 10.1002/cjp2.144 (PMC6966704; doi:10.1002/cjp2.144)
Supplement: Supplementary file 1 — Figure S1. Results of microsatellite and sequence analysis [file CJP2-6-12-s001.pdf]

Unravelling the genetic causes of mosaic islet morphology in congenital hyperinsulinism

Houghton, Banerjee *et al J Pathol Clin Res*, DOI 10.1002/cjp2.144

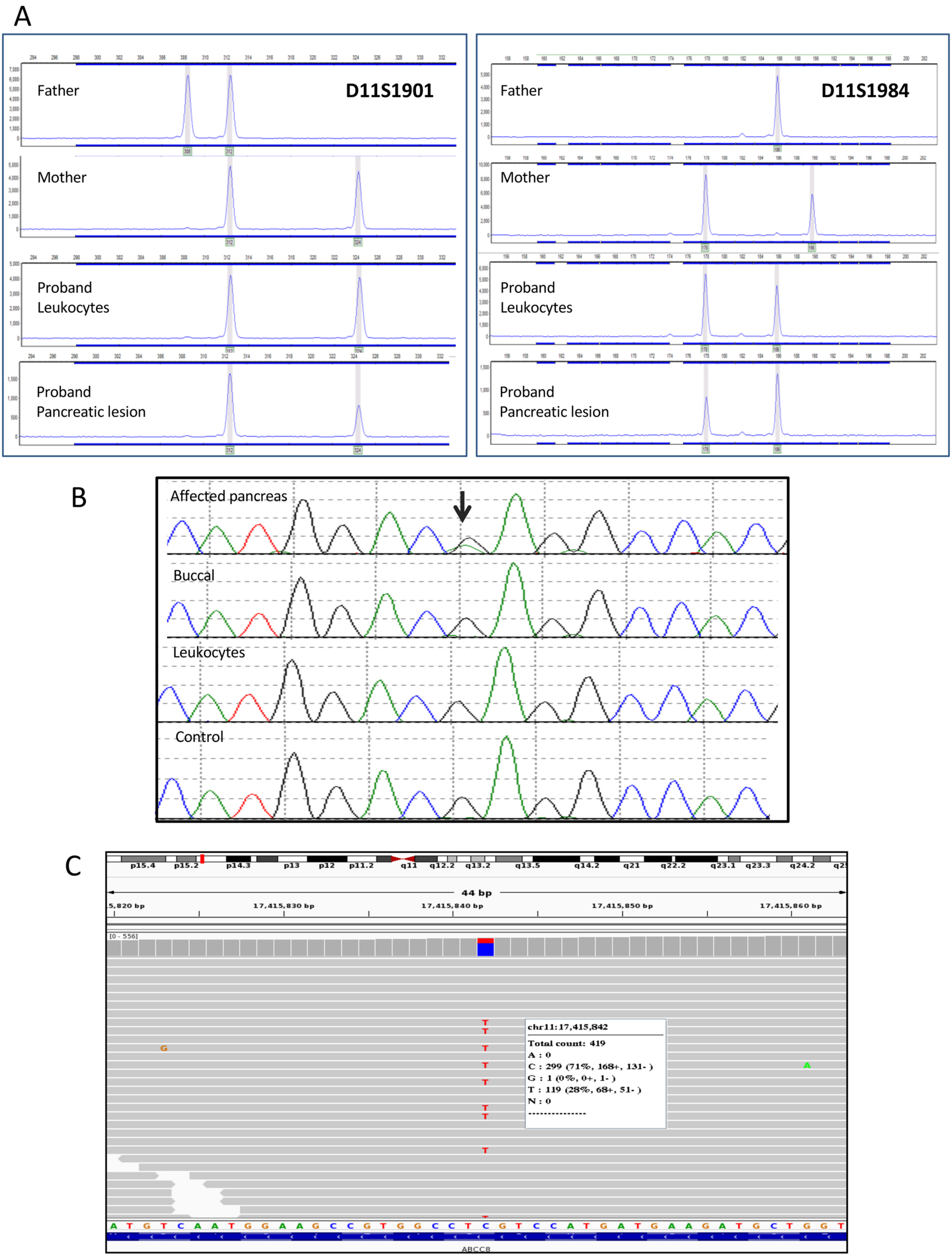

Figure S1. Results of microsatellite and sequence analysis. (A) Electropherograms demonstrating the results of microsatellite analysis of two informative markers on chromosome 11 (D11S1901 and D11S1984) in the proband (leukocytes and affected pancreatic tissue) and her parents' leukocyte DNA. Data for the 10 additional markers are not shown. The x-axis indicates the product size (bp), and the y-axis the product quantity (arbitrary units). The results illustrate mosaic loss of maternal heterozygosity for the two markers. (B) Sanger sequence electropherograms showing the p.(Glu1507Lys) c.4519G>A ABCC8 mutation in DNA extracted from the affected pancreatic tissue. Sequence traces showing the same position in DNA extracted from buccal cells and leukocytes from the patient are also provided along with a trace from an unaffected control. (C) An integrative genomics viewer screenshot showing the sequence reads (grey bars) mapping to 44 bp of exon 37 of ABCC8 on chromosome 11. The reference nucleotide sequence of the reverse strand is provided under the sequence reads. The substitution at Chr11(GRCh37):g.17415842C>T (NM_001287174.1c.4519G>A) resulting in the p.(Glu1507Lys) mutation is shown together with the allele counts.
